# Adipocytes Directly Affect Coronary Artery Disease Pathogenesis via Induction of Adipokine and Cytokine Imbalances

**DOI:** 10.3389/fimmu.2019.02163

**Published:** 2019-09-19

**Authors:** Olga Gruzdeva, Evgenya Uchasova, Yulia Dyleva, Daria Borodkina, Olga Akbasheva, Larisa Antonova, Vera Matveeva, Ekaterina Belik, Sergei Ivanov, Anton Sotnikov, Kirill Kozyrin, Natalia Brel, Maxim Sinitsky, Victoria Karetnikova, Alexander Kokov, Evgenya Bychkova, Tamara Pecherina, Olga Barbarash

**Affiliations:** ^1^Federal State Budgetary Institution, Research Institute for Complex Issues of Cardiovascular Diseases, Kemerovo, Russia; ^2^Federal State Budget Educational Institution of Higher Education, Kemerovo State Medical University of the Ministry of Healthcare of the Russian Federation, Kemerovo, Russia; ^3^Regional Center for Diabetes, Autonomous Public Healthcare Institution of the Kemrovo Region, Kemerovo Regional Clinical Hospital Named After S.V. Beliyaev, Kemerovo, Russia; ^4^Federal State Budget Educational Institution of Higher Education, Siberian State Medical University of the Ministry of Healthcare of the Russian Federation, Tomsk, Russia

**Keywords:** visceral obesity, epicardial adipose tissue, insulin resistance, inflammation, cytokine, adipokine

## Abstract

This study aimed to investigate the adipokine and cytokine profiles of adipocytes from epicardial and subcutaneous adipose tissues in interconnection with the visceral adipose tissue area and the biochemical and clinical characteristics of patients with coronary artery disease. We assessed 84 patients with coronary artery disease (65 men, 19 women) and divided them into two groups based on the presence of visceral obesity. We sampled epicardial and subcutaneous adipose tissues from the patients with visceral obesity. We then cultured the adipocytes and evaluated their adipokine profiles and pro-inflammatory activity. Results show that the mRNA expression of adiponectin in cultures of epicardial adipocytes from patients with and without visceral obesity was lower than that in subcutaneous adipocytes. Moreover, adiponectin mRNA expression in cultures of subcutaneous and epicardial adipocytes from patients with visceral obesity was lower than that in patients without obesity. For leptin, the reverse pattern was observed, with expression higher in cultures of epicardial adipocytes than in subcutaneous adipocytes and higher in epicardial adipocytes from patients with visceral obesity than in those from subjects without visceral obesity. In addition, in epicardial adipocytes, increased expression of proinflammatory cytokine genes (*IL6, TNF*) was observed compared with that in subcutaneous adipocytes. In contrast, expression of *IL10* was higher in cultures of subcutaneous adipocytes than in epicardial adipocytes. The epicardial adipose tissue area was associated with the presence of higher levels of leptin and TNF-α within adipocytes and serum, increased lipid and carbohydrate metabolism. Coronary artery disease, in the context of the status of epicardial adipocytes, can be characterized as “metabolic inflammation,” suggesting the direct involvement of adipocytes in pathogenesis through the development of adipokine imbalances and activation of proinflammatory processes.

## Introduction

Cardiovascular disease (CVD) is the leading cause of disability and mortality worldwide (1). In 2013, more than 17.3 million people died from CVD, an increase of 40.8% from 1990 (2). This increase was driven by a change in the number of deaths attributed to the aging population and the negative impact of cardiovascular risk factors, including obesity (3). Total body fat comprises accumulated fat in both the subcutaneous and visceral depots (4). Visceral adipose tissue (VAT) surrounds the internal organs and is associated with cardiometabolic risk factors, regardless of the total fat mass (5, 6).

Previous publications have highlighted associations between ectopic fat deposits and cardiometabolic risks for and clinical manifestations of CVD (7). Epicardial adipose tissue (EAT) is visceral fat deposited around the heart, particularly around epicardial coronary vessels. Because of its proximity to the myocardium and the absence of fascia, epicardial fat may directly affect the coronary arteries and myocardium (8). Several studies have shown that excessive amounts of EAT are accompanied by a reduction in adiponectin synthesis, myocardial hypertrophy, fibrosis, and the apoptosis of cardiomyocytes, leading to ventricular arrhythmias (9). However, epicardial adipocytes under physiological conditions have specific cardioprotective functions that include absorbing excess free fatty acids (FFAs), acting as a source of energy during ischaemia, and synthesizing adiponectin and adrenomedullin, both of which have cardioprotective effects (10). In addition, there is evidence of differences in the expression of the adipokine, leptin, and cytokine genes at the mRNA level in epicardial and subcutaneous adipose tissue (SAT) (11–13).

The present study aimed to investigate the adipokine and cytokine profiles of adipocytes from the EAT and SAT and in the serum, as well as to determine their relationships with the visceral fat area and the biochemical and clinical parameters of patients with coronary artery disease (CAD).

## Materials and Methods

### Ethical Considerations

The study protocol was approved by the Local Ethics Committee of the Federal State Budgetary Institution Research Institute for Complex Issues of Cardiovascular Diseases and was developed in accordance with the WMA Declaration of Helsinki on Ethical Principles for Medical Research Involving Human Subjects, 2000 edition, and the GCP Principles in the Russian Federation approved by the Russian Ministry of Health (2003). All patients provided written informed consent.

### Study Subjects

Eighty-four patients with CAD, including 65 men and 19 women with a mean age of 61.9 years (range: 53.7–68.5 years) were included in the study (all women were postmenopausal), who underwent coronary artery bypass grafting. Diagnosis of CAD was achieved according to the criteria of the All-Russian Scientific Society of Cardiology (2007) and the European Cardiology Society (2013). Blood samples were taken from patients with and without VO for assessment of serum levels of adipokines, cytokines, and regulators of lipid and carbohydrate metabolism. All patients were administered standard antianginal and antiplatelet therapies.

The control group comprised 30 male patients who did not have CVD and whose mean age was 58.42 years (range: 52.2–71.1 years). The ages of the patients in the control group were comparable with those in the study group (*p* = 0.64).

### Imaging Assessments

**Figure 1:**
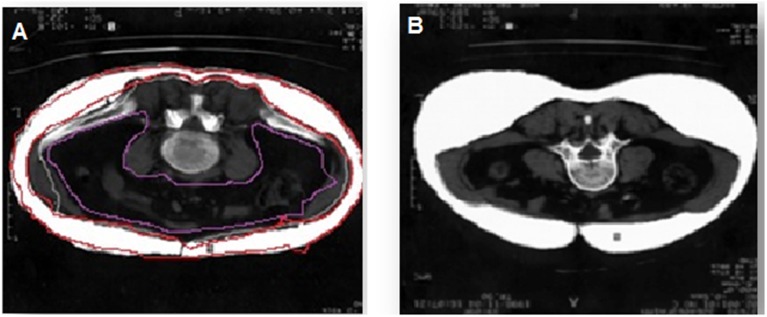
Diagnosis of visceral obesity via multi-spiral computed tomography. **(A)** Patient with obesity, **(B)** patient without obesity.

Multi-spiral computed tomography (MSCT) was performed on a Siemens Somatom 64 computed tomography scanner (Siemens Healthcare, Erlangen, Germany) for measurement of the visceral fat area and subcutaneous fat area to confirm the presence of visceral obesity (VO). A visceral fat area >130 cm^2^ and a VAT-to-SAT ratio at the level of the umbilicus of ≥0.4 were used as diagnostic cut-off values for VO (14) (Figure 1). Of the 84 patients, 54 had VO.

**Figure 2:**
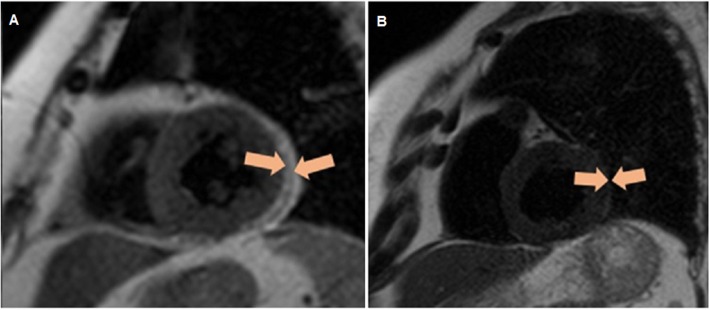
Quantitative assessment of the thickness of epicardial adipose tissue along the anterior wall of the right ventricle, as well as the thickness of the epicardial adipose tissue (orange contour) along the posterior wall of the left ventricle via magnetic resonance imaging. **(A)** Patient without obesity, **(B)** patient with obesity.

Determination of the thickness of EAT was performed by magnetic resonance imaging (MRI) on an Exelart Atlas 1.5-T MR imager (Toshiba, Tokyo, Japan). Measurements were carried out on images oriented along the short axis of the heart. EAT thickness was measured at three points along the anterior wall of the right ventricle, and the average value was then calculated (Figure 2).

The body weight in kilograms, height in meters, waist circumference (WC) in centimeters, and hip circumference (HC) in centimeters were measured in each subject, and the waist-to-hip ratio (WHR) and body mass index (BMI) in kilograms per square meter were calculated.

### Adipocyte Extraction and Culture

From each patient, 3–5 g of SAT and EAT biopsies were collected during coronary artery bypass surgery for isolation of adipocytes from freshly harvested tissues. EAT samples were obtained from fat depots located mainly around the right heart chambers, and SAT samples were obtained from the subcutaneous tissue in the region of the inferior mediastinum. The adipose tissue samples were placed in Hanks' Balanced Salt Solution (Merck KGaA, St. Louis, MO, USA) containing penicillin (100 U/L), streptomycin (100 mg/mL), and gentamicin (50 μg/mL). Adipocytes were isolated from adipose tissues under sterile conditions in a laminar flow hood (BOV-001-AMS MZMO, Millerovo, Russia) using the protocol developed by Carswell et al. (15). Adipose tissue samples (1–2 mm^3^) were incubated in a collagenase solution (0.5 mg/mL) (Thermo Fisher Scientific, Waltham, MA, USA) containing 200 nM adenosine (Merck KGaA) in a water bath at 37°C for 30 min. Then, the adipocytes were poured through a Falcon™ 100-μm sterile mesh (Thermo Fisher Scientific) and washed with Gibco® M199 culture medium (Thermo Fisher Scientific) containing 1% 4-(2-hydroxyethyl)-1-piperazineethanesulfonic acid buffer (Thermo Fisher Scientific), 1% l-glutamine with penicillin and streptomycin (Thermo Fisher Scientific), 0.4% amphotericin B (Thermo Fisher Scientific), and glucose at a final concentration of 5 mmol/L, supplemented with 10% fetal bovine serum (Thermo Fisher Scientific). The volume of the liquid containing the cells was adjusted to 5 mL, and the cells were centrifuged for 2 min at 200 × g. Isolated adipocytes were placed in a separate tube, and the volume was adjusted to 1 mL with culture medium. Adipocytes were counted in a Goryaev's chamber, and cell viability was evaluated as described previously (16). Adipocytes (20 × 10^5^) were seeded into a 24-well-plate (Greiner Bio One International GmbH, Kremsmünster, Austria), and the volume in each well was adjusted to 1 mL with culture medium. Incubated for 24 h at a temperature of (37 ± 1) °C in an atmosphere of 5% CO_2_ and 10% oxygen. After 24 h, adipokine and cytokine profiles were analyzed.

### RNA Extraction

Before RNA extraction, all work surfaces and laboratory equipment were treated with RNase*Zap*™ RNase Decontamination Solution (Invitrogen, Carlsbad, CA, USA). Total RNA purification from isolated adipocytes was performed using the commercial RNeasy® Plus Universal Mini Kit (Qiagen, Hilden, Germany) according to the manufacturer's protocol with some modifications described previously (17). Briefly, not more than 5 × 10^5^ cells were washed with phosphate-buffered saline (PBS) and homogenized with 750 μL of QIAzol™ Lysis Reagent (Qiagen). Homogenate was centrifuged at 12,000 × g for 10 min at 4°C. The upper fat monolayer was completely discarded and 75 μL of gDNA Eliminator Solution (Qiagen) and 150 μL of chloroform were added to each tube; the samples were then centrifuged at 12,000 × g for 30 min at 4°C. The clean upper aqueous phase was transferred into new tube, admixed with 1.5 volumes of 95% ethanol, and processed for binding, washing, and elution of total RNA according to the protocol. Extracted RNA was stored at −70°C.

The quantity and quality of purified RNA were assessed using a NanoDrop 2000 Spectrophotometer (Thermo Fisher Scientific) by measuring the light absorbance at 280 nm, 260 nm, and 230 nm and calculating the 260/280 (A_260/280_) and 260/230 (A_260/230_) ratios. The integrity of the RNA was determined by electrophoresis in agarose gel, followed by visualization using the Gel Doc™ XR+ System (Bio-Rad, Hercules, CA, USA).

### cDNA Synthesis

Single-stranded cDNA was synthesized using the High-Capacity cDNA Reverse Transcription Kit (Applied Biosystems, Foster City, CA, USA) on a Veriti™ 96-Well Thermal Cycler (Applied Biosystems). Each 20-μL reverse transcription mix contained 2 μL of RT buffer, 0.8 μL of 100 nM dNTP mix, 2 μL of random primers, 1 μL of reverse transcriptase, 1 μL of RNase inhibitor, 3.2 μL of nuclease-free water, and 10 μL of RNA template (50 ng of RNA + nuclease-free water). Reverse transcription was performed using the program suggested by the kit's manufacturer.

The quantity and quality of synthesized cDNA were assessed using a NanoDrop 2000 Spectrophotometer. Samples were stored at −20°C.

### Gene Expression Analysis

Expression of the adiponectin, leptin and cytokines *IL6, TNF, IL10* genes was evaluated by quantitative real-time polymerase chain reaction (qPCR) using TaqMan™ Gene Expression Assays (Applied Biosystems) on a ViiA 7 Real-Time PCR System (Applied Biosystems). Each 20-μL reaction mix contained 10 μL of TaqMan™ Gene Expression Master Mix (Applied Biosystems), 1 μL of TaqMan™ Gene Expression Assay (Applied Biosystems), and 9 μL of cDNA template (100 ng of cDNA + nuclease-free water) and was amplified under the following thermal cycling conditions: 2 min at 50°C, 10 min at 95°C, and 40 cycles of 15 s at 95°C and 1 min at 60°C. As a negative control, we used 20 μL of reaction mix with no cDNA template. For each sample and negative control, three technical replicates were prepared.

Normalization of the PCR results was carried out using three reference genes, *ACTB, GAPDH*, and *HPRT1*, in accordance with currently accepted recommendations (18). To assess the effectiveness of PCR, amplification graphs and standard curves were analyzed using QuantStudio™ Real-Time PCR Software v.1.3 (Applied Biosystems). The expression of the studied genes (normalized quantification ratio, NRQ) was calculated by the Pfaffl method and is represented on a logarithmic (log_10_) scale as the fold change compared to that in control samples. Gene expression experiments were performed in accordance with MIQE Guidelines (19, 20).

### Laboratory Assays

Levels of leptin and adiponectin in the culture medium from EAT and SAT adipocytes and in serum samples were measured using commercially available enzyme immunoassay kits (R&D Systems, Minneapolis, MN, USA) according to manufacturer's instructions. Each sample was tested twice, and intra-assay coefficients of variation (CVs) averaged 5.8%, with all standard curve correlation coefficients >0.998. The levels of interleukin (IL)-6, IL-10, and tumor necrosis factor-α (TNF-α) in the culture medium from EAT and SAT adipocytes and in serum samples were measured using commercially available enzyme-linked immunosorbent assays (ELISAs) (Bender MedSystems GmbH, Vienna, Austria) (CV: 7.03–8.99%).

Serum total cholesterol (TC), triglyceride (TG), very low-density lipoprotein cholesterol (VLDL-C), low-density lipoprotein cholesterol (LDL-C), high-density lipoprotein cholesterol (HDL-C), apolipoprotein (Apo)-A1, Apo-B, FFA, glucose, and glycated hemoglobin (HbA1c) levels were measured using enzymatic methods with end points on an automatic biochemical analyser (Konelab 30i; Thermo Fisher Scientific). Serum insulin and C-reactive peptide levels were measured using commercially available ELISA kits (Monobind, Lake Forest, CA, USA). Insulin sensitivity was evaluated based on insulin and fasting glucose levels using the homeostasis model assessment (HOMA) index, as follows: insulin level × glucose level/22.5. Insulin resistance (IR) was diagnosed when the HOMA-IR index was >2.77 (21).

### Statistical Analyses

Statistical analysis was performed using GraphPad Prism 6 (GraphPad Software, La Jolla, CA, USA) and Statistica software, version 6.1 (Dell Software, Inc., Round Rock, TX, USA). The Mann–Whitney *U*-test was used to compare non-normally distributed independent variables. Spearman's rank correlation coefficients were calculated to identify dependencies between variables. The data are presented as medians and 25th and 75th quartiles. Differences with values of *p* < 0.05 were considered statistically significant.

## Results

### Anthropometric and Clinical Data

Demographic and clinical data of the study patients are shown in Table 1. Patients were divided into two groups based on presence of VO: the first group included 54 patients with VO, and the second group included 30 without VO (Table 2). Patients in the two groups were comparable in age, presence of risk factors for CVD (i.e., hypertension, smoking, dyslipoproteinaemia, angina pectoris, and congestive heart failure), and myocardial infarction based on clinical history.

**Table 1:** Clinical and anamnestic characteristics of the study patients.

**Parameter**	**Patients, *n* = 84**	**%**
**Family history**		
Cardiovascular pathology	52	61.9
Type 2 diabetes mellitus	17	20.2
**Anamnesis**		
Smoking, *n*	45	53.7
Documented arterial hypertension, *n*	83	98.8
Hypercholesterolemia, *n*	63	75.0
Angina clinic	67	79.8
Prior myocardial infarction, *n*	17	20.2
Atrial fibrillation	3	3.6
History of stroke	7	8.3
Percutaneous coronary intervention	17	20.2
Coronary artery bypass surgery	2	2.4
**CHF**		
CHF I	23	27.4
CHF IIA–B	10	11.9
**Characteristic of the prevalence of atherosclerotic lesion**		
Atherosclerosis of the 1st coronary artery	16	19.1
Atherosclerosis of the 2st coronary artery	18	21.4
Atherosclerosis of the 3st coronary artery	50	59,5
Isolated lesions of the coronary arteries	21	25.0
Defeat several pools with stenoses <30%	34	40.5
Defeat several pools with stenosis of 30–50%	20	23.8
Defeat several pools with stenosis >50%	9	10.7
**Comorbidities**		
Chronic bronchitis	9	10.7
Bronchial asthma	2	2.4
Gout	2	2.4
Peptic ulcer disease in remission	6	7.1
Chronic cholecystitis	14	16.6
Chronic pyelonephritis	18	21.4
Varicose disease of lower extremities	19	22.6
**Treatment strategy/group of drugs**		
β-blockers	84	100.0
Angiotensin-converting enzyme inhibitors	74	88.1
Diuretics	37	44.0
Nitrates	17	20.2
Aspirin	84	100.0
Statins	84	100.0

*BMI, Body mass index; CHF, congestive heart failure*.

**Table 2:** Baseline clinical characteristics of the study patients, based on the presence of visceral obesity.

**Parameter**	**Patients with visceral obesity, *n* = 54**	**Patients without visceral obesity, *n* = 30**	***p***
Mean age (range), years	58.5 (53; 63)	56 (51.5;63.5)	0.71
Male gender, *n* (%)	45 (83.3)	20 (66.7)	0.613
Mean BMI (range), kg/m^2^	28.7 (17.1;39.1)	25.9 (18.3;38.4)	0.005
**Characteristic adipose tissue distribution**			
Abdominal fat area, cm^2^	541 (381;725)	357 (253;623)	0.00
VAT area (range), cm^2^	197(145;301)	108 (64;124)	0.00
SAT area (range), cm^2^	316 (201;501)	253 (159;498)	0.00
VAT/SAT	0.62 (0.60;0.72)	0.43 (0.24;0.50)	0.00
**Risk factors**			
Documented arterial hypertension, *n* (%)	54 (100.0)	29 (96.7)	0.207
Smoking, *n* (%)	33 (61.1)	12 (40.0)	0.109
Hypercholesterolemia, *n* (%)	43 (79.6)	20 (66.7)	0.25
Prior myocardial infarction, *n* (%)	11 (20.4)	6 (20.0)	0.21
PCI	11 (20.4)	6(20.0)	0.12
Stroke	5 (9.3)	2 (6.7)	
Angina	45 (83.3)	22 (73.3)	0.23
Percutaneous coronary intervention	11(20.4)	6 (20.0)	0.12
Coronary artery bypass surgery	2 (3.7)		
**CHF**			
CHF I	14 (25.9)	9 (30.0)	0.17
CHF IIA-B	6 (11.1)	4 (13.3)	0.74
CHF III	–	–	–

*BMI, Body mass index; WC, waist circumference; HC, hip circumference; WHR, waist-to-hip ratio; SAT, subcutaneous adipose tissue; VAT, visceral adipose tissue; CHD, coronary heart disease; CHF, congestive heart failure*.

### Adipokine and Cytokine Expression Levels in the SAT and EAT

**Figure 3:**
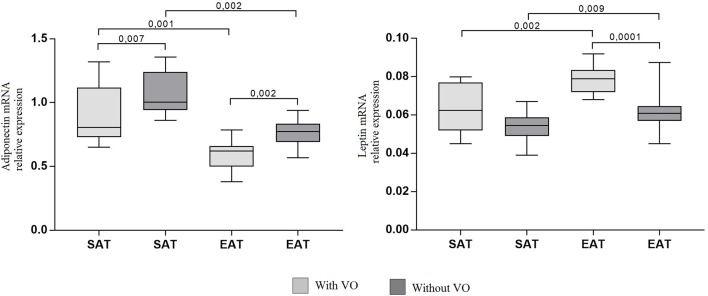
Expression of adipokine genes in adipocytes from the subcutaneous and epicardial fat depots. The data presented are the medians and the 25th and 75th quartiles. *p* < 0.05 for comparisons between the study groups.

The mRNA expression levels of adiponectin, leptin, and cytokines *IL6, TNF, IL10* were assessed in adipocyte cultures isolated from EAT and SAT. Expression of adiponectin in cultures of epicardial adipocytes from both patients with VO (*p* = 0.001) and without VO (*p* = 0.002) was lower than that in SAT adipocytes (Figure 3).

**Figure 4:**
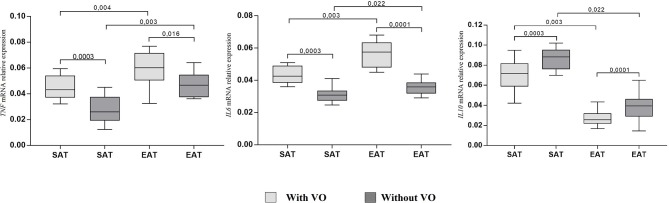
Expression of cytokine genes in adipocytes from the subcutaneous and epicardial fat depots. The data presented are the medians and the 25th and 75th quartiles. *p* < 0.05 for comparisons between the study groups.

At the same time, adiponectin mRNA expression in cultures of subcutaneous (*p* = 0.007) and epicardial (*p* = 0.002) adipocytes from patients with VO was lower than that in cultures from patients without obesity. For leptin, the reverse pattern was observed: expression was higher in cultures of epicardial adipocytes (*p* = 0.002, *p* = 0.009) than in those of subcutaneous adipocytes, and patients with VO (*p* = 0.002) exhibited higher expression of leptin mRNA in EAT adipocytes than in those without VO (*p* = 0.0001). In addition, in EAT adipocytes, increased expression of proinflammatory cytokines *IL6* and *TNF* was observed compared with that in SAT adipocytes (Figure 4). Moreover, expression in patients with VO was significantly higher than that in patients without VO, in both SAT and EAT cultures. In contrast, levels of *IL10* expression in SAT cultures were higher than those in EAT adipocytes. Between the two study groups, patients with VO were characterized by reduced expression of *IL10* (Figure 4).

The concentrations of adipokines and cytokines in the cultures of EAT and SAT adipocytes from patients with CAD are presented in Table 3. The leptin content was not significantly different between the cultures of epicardial and subcutaneous adipocytes. However, levels of leptin in patients with VO were significantly higher than those in patients without VO. Unlike leptin, levels of the protective molecule adiponectin were lower in EAT adipocytes than in SAT adipocytes, both in patients with VO (15% reduction) and without VO (21% reduction). At the same time, in patients without VO, levels of adiponectin in the culture media were significantly higher than those in patients with VO.

**Table 3:** Adipokine and cytokine levels in the adipocytes culture medium.

**Parameter**	**Subcutaneous adipose tissue**		**Epicardial adipose tissue**		***p***
	**Patients with VO** **1**	**Patients without VO** **2**	**Patients with VO** **3**	**Patients without VO** **4**	
Adiponectin (mg/mL)	17.44 (13.99;18.83)	21.03 (20.14;23.16)	14.77 (12.71;15.45)	19.39 (17.91;20.16)	*P*_1&2_ = 0.001 *P*_3&4_ = 0.0001 *P*_1&3_ = 0.017 *P*_2&4_ = 0.031
Leptin (pg/mL)	7.13 (6.18;8.03)	6.33 (5.55;6.99)	7.41 (6.95;8.17)	5.09 (3.99;6.28)	*P*_1&2_ = 0.003 *P*_3&4_ = 0.0023
IL-6 (pg/mL)	4.99 (3.19;5.97)	4.11 (3.74;5.28)	13.02 (8.37;14.93)	9.17 (8.48;11.31)	*P*_3&4_ = 0.001 *P*_1&3_ = 0.001 *P*_2&4_ = 0.002
TNF-α (pg/mL)	0.59 (0.47;0.71)	0.80 (0.68;0.91)	0.92 (0.74;1.02)	0.49 (0.39;0.56)	*P*_1&2_ = 0.014 *P*_3&4_ = 0.021 *P*_1&3_ = 0.011 *P*_2&4_ = 0.001
IL-10 (pg/mL)	7.14 (5.97;8.78)	8.21 (7.77;9.44)	6.04 (5.01;7.41)	7.32 (6.25;8.13)	*P*_3&4_ = 0.033 *P*_1&3_ = 0.037 *P*_2&4_ = 0.0019

*The data presented are the medians (25th quartiles, 75th quartiles). CAD, coronary artery disease; VO, visceral obesity; TNF-α, tumor necrosis factor-α; IL,interleukin*.

Levels of proinflammatory cytokines *TNF* and *IL6* were significantly higher in EAT adipocytes than in SAT adipocytes, whereas concentrations of the protective cytokine *IL10* were lower in EAT than in SAT cultures, among both patients with VO (25% reduction) and without VO (30% reduction). Moreover, higher levels of proinflammatory cytokines and lower levels of the anti-inflammatory *IL10* were noted in patients with VO compared to levels in patients without VO.

### Serum Adipokine and Cytokine Levels

Analysis of the sera from CAD patients with VO (Table 4) showed that serum adiponectin levels in patients with VO were 27.6% lower than those in patients without VO. Additionally, a 1.67-fold increase in leptin levels was observed in patients with VO compared to levels in CAD patients without VO.

**Table 4:** Adipokine and cytokine levels in the serum of patients with cardiovascular disease with and without visceral obesity.

**Parameter**	**Control group,** ***n* = 30**	**Patients with visceral obesity,** ***n* = 54**	**Patients without visceral obesity,** ***n* = 30**	***p***
	**1**	**2**	**3**	
Leptin (pg/mL)	6.9 (4.51, 9.75)	14.6 (11.2, 18.9)	8.7 (6.4, 9.9)	*p*_1&2_ = 0.01 *p*_2&3_ = 0.02
Adiponectin (mg/mL)	12.36 (7.30, 13.5)	8.1 (6.3, 10.8)	11.2 (8.7, 14.2)	*p*_1&2_ = 0.03 *p*_2&3_ = 0.01
IL-6 (pg/mL)	3.90 (2.80; 4.10)	11.24 (9.36; 14.94)	7.76 (2.7; 12.19)	*p*_1&2_ = 0.01 *p*_2&3_ = 0.05 *p*_1&3_ = 0.04
TNF-α (pg/mL)	1.2 (0.9, 1.4)	1.5 (1.0, 2.3)	1.0 (0.8, 2.1)	*p*_1&2_ = 0.04 *p*_1&3_ = 0.04 *p*_2&3_ = 0.03
IL-10 (pg/mL)	8.9 (7.4, 10.2)	3.7 (1.1, 4.8)	8.3 (6.8, 9.7)	*p*_1&2_ = 0.01 *p*_2&3_ = 0.01

*The data presented are the medians (25th quartiles, 75th quartiles). TNF, tumor necrosis factor; IL,interleukin*.

Serum TNF-α levels were 1.5-fold higher in patients with VO than in those without VO. Levels of IL-6 also increased 2.9 and 2 times in patients with VO and without VO, respectively, indicating higher levels in patients with VO than in those without VO. IL-10 levels were 2-fold lower in patients with VO than in patients without VO. Positive correlations between levels of leptin, TNF-α, and IL-10 in adipocytes and those in sera were evident, with correlation coefficients of 0.43 (*p* = 0.04), 0.53 (*p* = 0.02), and 0.50 (*p* = 0.04), respectively.

### Factors Associated With Lipid and Carbohydrate Metabolism

Compared with patients without VO, serum levels of atherogenic factors associated with lipid metabolism, namely, LDL-C, VLDL-C, Apo-B, TGs, and Apo-B/Apo-Al, increased significantly in patients with VO, whereas antiatherogenic fractions, namely, HDL-C and Apo-A1, were lower in patients with VO. Changes in carbohydrate metabolism were more pronounced in CAD patients with VO compared to those in patients without VO. IR was present in CAD patients with VO, who showed a 1.5-fold increase in the HOMA index compared with that in patients without VO. Hyperinsulinaemia was often present in patients with VO, and their C-reactive peptide levels were elevated compared with those in patients without VO. The two groups did not differ significantly with respect to glucose or HbA1c levels (*p* > 0.05).

### Correlations Between Adipose Tissue Area and Adipokine and Cytokine Levels

Some of the patients with VO had normal BMIs. Likewise, some of the patients with high BMIs and class II or III obesity did not have VO. In contrast to the SAT area, the EAT area was positively associated with higher levels of leptin and TNF-α in the adipocytes and serum, along with higher levels of factors associated with lipid and carbohydrate metabolism (Table 5). In contrast, serum adiponectin levels were associated with the SAT area (*r* = 0.47, *p* = 0.03).

**Table 5:** Correlations between the level of adipokines and cytokines in serum, in the adipocytes culture medium and the area of visceral adipose tissue.

	**Visceral adipose tissue areas, cm**^****2****^	
**Parameter**	***r***	***p***
Leptin in the adipocytes culture medium, pg/mL	0.48	0.02
TNF-α in the adipocytes culture medium, pg/mL	0.39	0.02
TNF-α in blood serum, pg/mL	0.52	0.01
Leptin in blood serum, pg/mL	0.38	0.02
TC in blood serum, mmol/l	0.32	0.04
VLDL-C in blood serum, mmol/l	0.44	0.03
TG in blood serum, mmol/l	0.37	0.04
FFAs in blood serum, mmol/l	0.32	0.03

*TNF, tumor necrosis factor; TC, total cholesterol; VLDL-C, very low-density lipoprotein cholesterol; TG, triglyceride; FFAs, free fatty acids; HOMA, homeostasis model assessment*.

## Discussion

Obesity is a leading risk factor associated with CVD, and VO is the factor with the strongest influence on the development of CVD. Cardiovascular risk increases by 2.75-fold for patients who present with VO and whose body weights are normal, and the risk of all-cause mortality increases by 2.08-fold compared with patients who do not have VO (22–26).

VAT is an active endocrine organ that releases adipokines, regulators of lipid and carbohydrate metabolism, and cytokines; hence, it can potentially contribute to atherogenesis. Moreover, VAT has unique metabolic functions. Compared with adipocytes from other fat depots, VAT adipocytes exhibit increases in β3-adrenoreceptor expression and function and a decrease in the number of insulin receptors, leading to an increase in lipid metabolism. Accumulations of FFAs and ceramide non-oxidized metabolites can stimulate the development of lipotoxic disorders, resulting in IR, dyslipidaemia, cardiomyopathy, and higher blood pressure (22). Within the VAT, the EAT may be of particular importance. EAT is a unique fat depot, the thickness, and metabolic activity of which correlate directly with the VAT area (27). The thickness of the EAT correlates directly with the severity of CAD in patients and with the thickness of the left ventricular myocardium (28).

VO is adversely associated with the development of cardiac pathology. We hypothesized that the adipokines and cytokines synthesized by EAT adipocytes from patients with VO and released into the blood were cardiovascular risk factors; hence, this study's primary focus was to investigate adipokine and cytokine profiles in adipocytes and in serum samples from CAD patients with VO. We found that EAT adipocytes had elevated leptin levels and reduced adiponectin levels compared with those in SAT adipocytes. The elevated leptin levels in the EAT and SAT adipocytes correlated directly with elevated concentrations in the serum.

In contrast to the present study, in the literature, there are several lines of evidence for the absence of a correlation between EAT-secreted cytokines and circulating cytokine levels. Early on, this contradiction was reported only for adiponectin; expression of adiponectin in EAT may or may not correlate with plasma concentrations, which are presumably derived from the SAT and most often decrease in obesity (29). Thus, we found that the expression of the adiponectin gene in cultures of epicardial adipocytes in both patients with and without VO was lower than that in subcutaneous adipocytes. This contradiction can be attributed to the nature of correlation analysis: a correlation between two variables may indicate the existence of a common cause, rather than a direct interaction between the two. After a chronic positive energy balance, the adipocytes in the VAT induce FFA uptake and accumulation. Expansion of VAT triggers the expression of proinflammatory adipokines, oxidative stress, and activation of the renin-angiotensin-aldosterone system. Hypertrophic but not hyperplastic adipocytes are associated with IR (30). Thus, the VAT becomes dysfunctional, dysregulating adipocyte apoptosis and increasing autophagy (30). Nevertheless, the accumulation of fat may saturate VAT capacity. The resultant failure of VAT to store triacylglycerides could result in ectopic deposition of toxic fatty acids species (i.e., diacylglycerol, ceramide) in additional adipose tissue such as the myocardium, leading to an increase in EAT thickness (31). Importantly, the amount of VAT correlates with the volume of the EAT, and thus, significant weight loss in obese patients has been associated with noteworthy reductions in EAT volume (32–34). In obesity, many changes occur, as EAT volumes substantially increase. These changes are often characterized by hypertrophy, failure to store triglycerides, increased lipolysis, and inflammation (35). Dysfunctional adipocytes express high levels of proinflammatory factors (i.e., IFN-γ) that enhance the proinflammatory response of infiltrated immune cells, such as dendritic cells, macrophages, T and B cells, and eosinophils (36). As EAT expands, it becomes hypoxic and dysfunctional (37) and is invaded by an increasing number of macrophages and T lymphocytes, resulting in a shift in its metabolic profile. The result is increased secretion of proinflammatory cytokines such as TNF-α, IL-6, and others that contribute to the inflammatory environment characteristic of atherogenesis (38). Consistent with this, in our study, increased expression of proinflammatory cytokines and decreased expression of an anti-inflammatory cytokine were observed in EAT adipocytes compared with levels in SAT adipocytes. All patients were found to have dyslipidaemia; however, in subjects with VO, levels of TGs, and FFAs were significantly higher than those in subjects without VO. It is possible that TGs act as a pathogenic trigger of adiposopathy in VAT and EAT.

Given the ability of proinflammatory cytokines to modulate the synthesis and secretion of adipokines, activation of inflammation may increase leptin levels and inhibit the protective effects of adiponectin in EAT adipocytes. The activation of inflammation in SAT adipocytes is associated with high levels of IL-10, and elevated leptin levels are accompanied by high adiponectin levels. Thus, analysis of the adipokine and cytokine profiles in this study suggested differences between EAT and SAT adipocytes.

Changes in the levels of leptin, adiponectin, TNF-α, IL-6, and IL-10 in the serum and in EAT adipocytes appeared to show similar patterns; hence, increases in levels of pathogenic factors and reductions in levels of protective factors were observed.

Importantly, the imbalance between disease-promoting and protective adipokines and cytokines in patients with VO was accompanied by more pronounced impairments in lipid and carbohydrate metabolism compared with those observed in patients without VO. Patients with VO showed more pronounced changes in adipokine and cytokine profiles of the serum and adipose tissue. However, changes in metabolism are not dependent on the thickness of the subcutaneous fat layer. The local and systemic imbalances of the adipokine and cytokine profiles in the patients with VO were accompanied by multivessel CAD with stenotic lesions that narrowed the vessels by 30–50%, angina pectoris functional class 2 or 3, increases in atherogenic fractions of serum lipids, and the development of IR. Adipokines and cytokines are apparently released directly into the coronary blood flow owing to the anatomical proximity of the EAT to the coronary arteries, which may have adverse consequences. Administration of TNF-α intra-arterially is accompanied by endothelium-dependent vasodilation and the initiation of inflammation in the vascular wall (39–42). The proatherogenic effects of TNF-α also include the promotion of leucocyte migration to the endothelium, increases in the synthesis of adhesion molecules and chemoattractants, and enhanced vascular permeability (38, 41). Based on the cytokine profiles observed in adipocytes, we conclude that the activation of inflammation in EAT adipocytes in patients with VO may be extremely important in CAD, with increases in TNF-α, IL-1, and IL-6 levels accompanied by decreases in IL-10 levels.

EAT adipocytes in CAD patients with VO may characterize “metabolic inflammation,” indicating that adipocytes are directly involved in the pathogenesis of CAD through development of an adipokine imbalance and activation of proinflammatory processes (43).

## Data Availability

The datasets analyzed in this manuscript are not publicly available. Requests to access the datasets should be directed to o_gruzdeva@mail.ru.

## Ethics Statement

The Ethics Committee of the Federal State Budgetary Institution, Research Institute for Complex Issues of Cardiovascular Diseases approved this study (Protocol No. 22 from 15.08.2017). All patients provided informed consent.

## Author Contributions

OG was principal investigator, study co-ordinator and investigator, participated in all stages of recruitment of the patients and in analysis of the data, drafted, and critically reviewed the manuscript. OA and VK were study co-ordinator and investigator, participated in all stages of recruitment of the patients and in analysis of the data, drafted, and critically reviewed the manuscript. EU, AK, TP, EBe, LA, VM, and YD were study investigator, participated in all stages of recruitment of patients and critically reviewed the manuscript. SI, AS, and KK obtained bioptaps of adipose tissue of various localization during coronary bypass surgery. OB was principal investigator. EBy was study investigator, participated in all stages of recruitment of patients and collected the data. All other study investigators conducted the study and collected the data. All authors read and approved the final manuscript.

### Conflict of Interest Statement

The authors declare that the research was conducted in the absence of any commercial or financial relationships that could be construed as a potential conflict of interest.
